# ABT-263 exerts a protective effect on upper urinary tract damage by alleviating neurogenic bladder fibrosis

**DOI:** 10.1080/0886022X.2023.2194440

**Published:** 2023-05-08

**Authors:** Yibo Wen, Junwei Wu, Qingsong Pu, Xiangfei He, Junkui Wang, Jinjin Feng, Yanping Zhang, Feng Si, Jian Guo Wen, Jinghua Yang

**Affiliations:** a Department of Urology, The First Affiliated Hospital of Zhengzhou University, Zhengzhou, P.R. China; b Clinical Systems Biology Laboratories of the First Affiliated Hospital of Zhengzhou University, Zhengzhou, P.R. China; c The Academy of Medical Science, Zhengzhou University, Zhengzhou, P.R. China; d Bladder Structure and Function Reconstruction Henan Engineering Laboratory, The First Affiliated Hospital of Zhengzhou University, Zhengzhou, P.R. China; e Department of Urology, The First People’s Hospital of Longquanyi District, Chengdu, P.R. China; f Department of Ultrasound, The First Affiliated Hospital of Zhengzhou University, Zhengzhou, P.R. China; g Department of Urology, The First Affiliated Hospital of Xinxiang Medical College, Xinxiang, P.R. China

**Keywords:** ABT-263, neurogenic bladder, fibrosis, apoptosis, kidney

## Abstract

This study investigated the mechanism of action of ABT-263 in the treatment of neurogenic bladder fibrosis (NBF)and its protective effects against upper urinary tract damage (UUTD). Sixty 12-week-old Sprague-Dawley (SD) rats were randomly divided into sham, sham + ABT-263 (50 mg/kg), NBF, NBF + ABT-263 (25 mg/kg, oral gavage), and NBF + ABT-263 (50 mg/kg, oral gavage) groups. After cystometry, bladder and kidney tissue samples were collected for hematoxylin and eosin (HE), Masson, and Sirius red staining, and Western Blotting (WB) and qPCR detection. Primary rat bladder fibroblasts were isolated, extracted, and cultured. After co-stimulation with TGF-β1 (10 ng/mL) and ABT-263 (concentrations of 0, 0.1, 1, 10, and 100 µmol/L) for 24 h, cells were collected. Cell apoptosis was detected using CCK8, WB, immunofluorescence, and annexin/PI assays. Compared with the sham group, there was no significant difference in any physical parameters in the sham + ABT-263 (50 mg/kg) group. Compared with the NBF group, most of the markers involved in fibrosis were improved in the NBF + ABT-263 (25 mg/kg) and NBF + ABT-263 (50 mg/kg) groups, while the NBF + ABT-263 (50 mg/kg) group showed a significant improvement. When the concentration of ABT-263 was increased to 10 µmol/L, the apoptosis rate of primary bladder fibroblasts increased, and the expression of the anti-apoptotic protein BCL-xL began to decrease.ABT-263 plays an important role in relieving NBF and protecting against UUTD, which may be due to the promotion of myofibroblast apoptosis through the mitochondrial apoptosis pathway.

## 1. Introduction

Neurogenic bladder (NB) refers to the dysfunction of lower urinary tract storage and urination due to damage to the central or peripheral nervous system, resulting in a series of lower urinary tract symptoms (LUTS) and complications [1]. NB can lead to different LUTS, such as dysuria, urinary incontinence, and urinary retention, as well as complications, such as vesicoureteral reflux (VUR), ureteral dilatation, and hydronephrosis [2]. Upper urinary tract damage(UUTD) is the most serious complication of NB because it can cause kidney failure requiring dialysis or kidney transplantation [3]. Therefore, the main goal of NB treatment is to protect upper urinary tract function and ensure that bladder pressure during storage and voiding is within a safe range. Bladder compliance (BC) reflects the relationship between changes in bladder volume (BV) and detrusor pressure [4]. If BC is reduced due to neurogenic lesions, intravesical pressure can increase excessively with an increase in urine in the bladder. A persistent increase in bladder pressure causes UUTD [5]. Studies have shown that the greater the degree of bladder fibrosis, the worse the BC and the higher the detrusor leakage point pressure, which will increase the possibility of UUTD. Reducing the degree of bladder fibrosis increases BC [6,7]. Therefore, the degree of NB fibrosis (NBF) is closely related to UUTD.

It is well known that the TGF-β1 signaling pathway plays a key role in the fibrosis process of organs, including the urinary bladder [8,9]; however, TGF-β1 is also involved in very important physiological functions in the body, such as cell growth, differentiation, and apoptosis. Therefore, targeting TGF-β1 is not an ideal point of action for anti-fibrosis treatment [10].

The ability of myofibroblasts to synthesize and release the extracellular matrix (ECM) components is greatly enhanced, which can promote the formation of large collagen bundles that together make the tissue stiffer. Moreover, myofibroblasts have a strong resistance to apoptosis, continuously release ECM, and aggravate fibrosis [11,12]. Therefore, targeting myofibroblasts may be an effective strategy to alleviate NBF and has attracted the interest of many researchers [13–15].

ABT-263 is a known BH3 mimetic drug that effectively binds to the BH3 domain of the anti-apoptotic member of BCL-2 and can induce apoptosis through a mitochondrial apoptosis mechanism [16]. Myofibroblast apoptosis can prevent and reverse the progression of fibrotic diseases such as systemic sclerosis. Studies have shown that ABT-263 can alleviate the progression of skin fibrosis [17], pulmonary fibrosis [18], biliary fibrosis [19], and myocardial fibrosis [20] by promoting myofibroblast apoptosis. However, there are no reports on the therapeutic effect of ABT-263 in NBF, especially in severe NBF with UUTD. Therefore, this study investigated ABT-263 in the treatment of NBF, its protective effect on the upper urinary tract, and its potential mechanism of action.

## 2. Materials and methods

### 2.1. Experimental animals

Sixty 12-week-old female Sprague-Dawley (SD) rats weighing 183 ± 15 g were provided by the Henan Provincial Laboratory Animal Center (Zhengzhou, China) under the animal license number SCXK (Yu) 2017-0001. The rats were housed in a Specific Pathogen Free (SPF) grade animal room with an independent ventilation system, a 12-h light and dark cycle, 22 ± 1 °C, humidity of 45–55%, and free access to water and food. The experimental protocol was approved by the Ethics Committee of the First Affiliated Hospital of Zhengzhou University (approval number: 2018-KY-86). All animal experiments were performed in accordance with protocols reviewed and approved by the American Experimental Management Committee (IACUC).

### 2.2. Preparation of ABT-263

ABT-263 was purchased from Adooq Bioscience (A10022-5; Irvine, CA, USA). ABT-263 was formulated in 10% ethanol, 30% polyethylene glycol 400, and 60% Phosal 50 PG (a dispersion of 50% phosphatidylcholine in propylene glycol) and administered by oral gavage *in vivo*. ABT-263 was prepared in solvents with concentrations of 0, 0.1, 1, 10, and 100 µmol/L in dimethyl sulfoxide (DMSO) for *in vitro* experiments.

### 2.3. Animal model and grouping

Fifty 12-week-old SD rats were randomly divided into sham (*n* = 8), sham + ABT-263 (50 mg/kg, gavage) (*n* = 10), NBF (*n* = 11), NBF + ABT-263 (25 mg/kg, gavage) (*n* = 9), and NBF + ABT-263 (50 mg/kg, gavage) (*n* = 12) groups [17]. NBF was induced by bilateral L6 + S1 spinal nerve amputation as reported in our previous publication [21]. Briefly, the rats were anaesthetized by isoflurane inhalation, and the lumbar 6 (L6) lamina was removed, exposing the bilateral L6 nerves and cauda equina, which were completely transected to create paralysis of the bladder. After adequate hemostasis, the wound was sutured using 4–0 silk. Ten rats died of infection or renal failure before the end of the observation period. Three rats were assigned to the sham + ABT-263 group (25 mg/kg), two rats were assigned to the NBF + ABT263 group (50 mg/kg), and five rats were assigned to the NBF group. Consequently, the final number was eight in the sham group, ten in the sham + ABT-263 group (50 mg/kg, gavage), six in the NBF group, six in the NBF + ABT-263 group (25 mg/kg, gavage), and ten in the NBF + ABT-263 group (50 mg/kg, gavage). We started ABT-263 administration (twice a week) for four weeks on the rats three months after the surgical procedure, when NBF was successfully established. In detail, the sham and NBF groups were gavaged with saline, sham + ABT-263 and NBF + ABT263 groups were gavaged with 50 mg/kg ABT-263. The other NBF + ABT-263 group was administered 25 mg/kg ABT-263 twice a week for four weeks, as described by Chen et al. [22]. Renal ultrasonography and cystometry were performed, and bladder and kidney tissue samples were collected for hematoxylin and eosin (HE), Masson, and Sirius red staining, and Western blotting (WB) and qPCR detection. Blood was drawn from the inferior vena cava to determine serum creatinine (SC) and blood urea nitrogen (BUN) levels.

### 2.4. Isolation, identification, and culture of primary rat bladder fibroblasts

Three one-week-old SD rats were anesthetized, and the bladder tissue was peeled off under aseptic conditions, placed into a petri dish containing phosphate-buffered saline (PBS), washed, and transferred to a new tube. After adding an appropriate amount of 0.1% trypsin and collagenase mixed enzyme solution, the tissue samples were placed in a constant temperature shaker at 37 °C for 30 min of digestion. Digestion was terminated with DMEM/F12 complete medium containing 10% fetal bovine serum (FBS). The filtrate was collected through a 100 µm stainless steel mesh and centrifuged at 1500 rpm for 5 min. The pellet was resuspended in 5 mL of complete medium, inoculated into a 25 cm^2^ culture flask, and placed in a 5% CO_2_ incubator at 37 °C after observation under a microscope. Cell identification was performed by immunofluorescence using α-SMA antibody (48938S; CST, Danvers, MA, USA).

### 2.5. Ultrasound and urodynamic examination

The anterior and posterior diameters of the renal pelvis were measured using a multifunctional Doppler ultrasound (LOGIQ E9; GE, Boston, MA, USA) and a 4–15 MHz probe to evaluate hydronephrosis. Rats were anaesthetized by intraperitoneal injection of urethane [23] at a concentration of 20% and a dose of 1 g/kg, and a blunt epidural catheter with a diameter of 1 mm was smeared with a paraffin oil globule and inserted into the bladder for cytometry, as described in our previous publication [24]. Bladder pressure curves and basal bladder pressure, P_ves.thre_(bladder pressure just before voiding), P_ves.leak_(pressure in the bladder when filling incontinence occurs), P_ves.max_ (maximum pressure during voiding), voiding interval, and time to first leakage were recorded.

### 2.6. Tissue weight and pathological staining

The bladder was carefully stripped of adipose tissue and the outer membrane, washed with PBS, dried with filter paper, weighed with an analytical balance, and photographed. Bladder and kidney tissues were soaked in 4% paraformaldehyde overnight, dehydrated in a gradient, embedded in paraffin, and cut into 4 µm sections. HE and Masson staining were performed according to standard procedures for observation and scanning. Sirius red staining was photographed and examined under an upright polarized light photographic microscope, and the images were analyzed using Image-Pro Plus 6.0.

### 2.7. Quantitative real-time polymerase chain reaction (qRT-PCR)

The total RNA of bladder tissue was extracted using the Trizol method (B511311; Sangon Biotech, Shanghai, China) and stored at −80 °C. A NanoDrop 2000 UV–Visspectrophotometer (Thermo Fisher Scientific, Waltham, MA, USA) was used to determine the concentration and quality of total RNA. The aMuLV First-Strand cDNA Synthesis Kit (B532435; Sangon Biotech) was used for the reverse transcription of total RNA. An Applied Biosystems 7500 Sequence Detection System was used to determine RNA levels with a 2 × SG Fast qPCR Master Mix (Low Roxx) kit (B639272; Sangon Biotech). Each sample was analyzed in triplicate.

GAPDH served as a control and the data were analyzed using the 2 ^−(ΔCt)^ method. We conducted qRT-PCR and an Applied Biosystems 7500 Sequence Detection System was used to detect the levels of col1, col3, and FN. The sequences of all primers used are listed below.

R-GAPDH-FCAAGTTCAACGGCACAGTCAAG

R-GAPDH-R ACATACTCAGCACCAGCATCAC

R-collagen1-F GATGGACTCAACGGTCTCCC

R-collagen1-R CGGCCACCATCTTGAGACTT

R-collagen3-F CTTCTCACCCTGCTTCACCC

R-collagen3-R GGGCAGTCTAGTGGCTCATC

R-FN-FCCCACCAGCCTGCTCATC

R-FN-RGGGCTATTTCCTCCTGTCTCTC

### 2.8. Detection of cell proliferation and apoptosis

Primary bladder fibroblasts were plated in two 96-well plates at 1 × 10^3^ per well, placed in a 5% CO_2_ cell incubator at 37 °C overnight, and the medium was changed to contain 10 ng/mL of TGF-β1(100-21C; Peprotech China, Suzhou, Jiangsu Province, China). After stimulation with 10 ng/mL TGF-β1 for 24 h, the medium was replaced with ABT-263 at a concentration of (0–100 µm) and incubated for another 24 h. CCK-8(CK04-01; (CCK-8: Dojindo Molecular Technologies Inc., Rockville, MD, USA) reaction solution was added to 96-well plates (10 µL/well) and then placed in a cell incubator for 1 h. Finally, the absorbance value was measured, and the apoptosis rate of the cells was detected by annexin V/PI double staining using flow cytometry.

### 2.9. Immunofluorescence staining of cells

According to the standard procedure [25] for cellular immunofluorescence, anti-α-SMA antibody was used as the primary antibody and incubated at 4 °C overnight. The diluted fluorescent secondary antibody Alexa Fluor 488 (A32731; Thermo Fisher Scientific) was added dropwise and incubated at 20–37 °C for 1h in a wet box. DAPI was added dropwise and the mixture was incubated in the dark for 5 min. After mounting the slides on the collected images, they were observed under a fluorescence microscope.

### 2.10. Western blotting

The total protein of the tissue or cells was extracted using the BCA method to determine the protein concentration, and the protein was denatured to prepare a sample. SDS-PAGE was performed, and the membrane was transferred, blocked with 5% milk on a shaker for 1 h, and incubated with the following primary antibodies: col1 (SC-393573), col3 (SC-271249), FN(SC-8422)(Santa Cruz Biotechnology, Dallas, TX, USA); TGF-β1 (21898-1-AP; Proteintech, Wuhan, China); α-SMA (CST-48938S); BCL-xL (CST-2764S, CST, Danvers, MA, USA); GAPDH, (CST-8884, CST, Danvers, MA, USA). The membrane was shaken at 4 °C overnight, washed, incubated with the secondary antibody for 1 h at room temperature, washed, and exposed.

### 2.11. Statistical methods

All data are expressed as mean ± standard error of the mean (SEM). GraphPad Prism software (version 6.0) was used for the statistical analysis. Comparisons between the two groups were performed using *t-*tests. One-way analysis of variance (ANOVA) was used to compare the means of multiple samples. *P* values <0.05 were considered statistically significant.

## 3. Results

### 3.1. ABT-263 treatment can alleviate neurogenic hydronephrosis in rats

**Figure 1.**
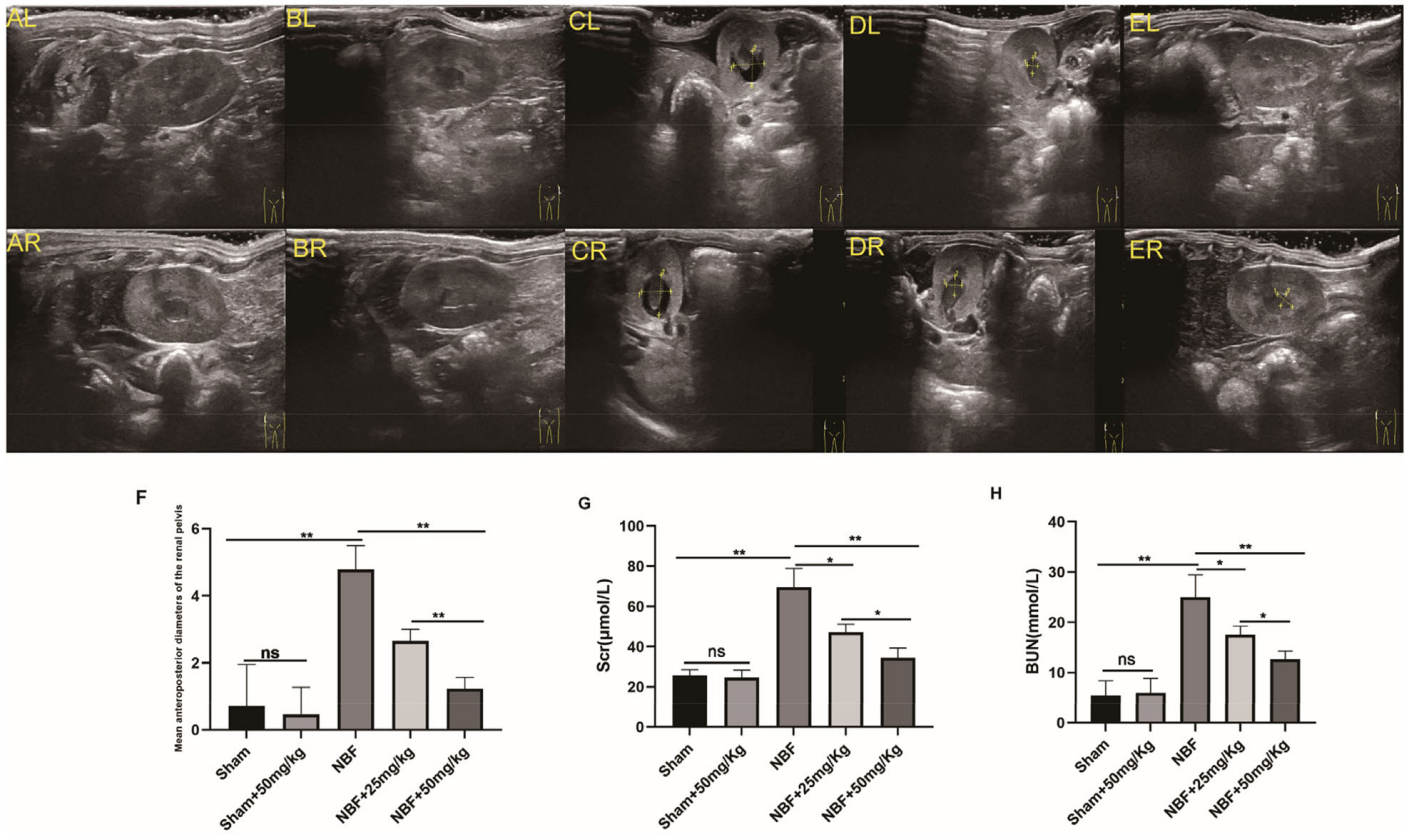
Ultrasound images of different groups show the difference in diameter between the anterior and posterior renal pelvis.ALandAR: left and right kidneys in the sham group; there was no obvious hydronephrosis in the renal pelvis. BLandBR: left and right kidneys in the sham + ABT-263 (50 mg/kg) group; there was no notable hydronephrosis in the renal pelvis. CLandCR: left and right kidneys in the neurogenic bladder fibrosis (NBF) group andhydronephrosis in the renal pelvis. DLandDR: left and right kidneys in the NBF + ABT-263 group (25 mg/kg); compared with the NBF group, hydronephrosis in the renal pelvis was significantly reduced. ELandER: left and right kidneys in the NBF + ABT-263 group (50 mg/kg). Compared with the NBF + ABT-263 group (25 mg/kg), hydronephrosis in the renal pelvis was significantly reduced. The yellow arrow indicates the hydronephrosis area. f: Statistical histograms of the anterior and posterior diameters of the renal pelvis (the anterior and posterior diameters of the left and right kidneys were added together and then averaged); gandh: Statistical histograms of serum creatinine (SC) and blood urea nitrogen (BUN) levels. Data are expressed as the mean ± SEM; ns *p* > 0.5, *p < 0.01, **p < 0. 001. All experiments were repeated three times.

There was no statistically significant difference between the sham group and the sham + ABT-263 (50 mg/kg) group in SC, BUN, and anterior and posterior diameters of the renal pelvis (Figure 1). The SC, BUN, and anterior and posterior diameters of the renal pelvis in the NBF group were significantly increased compared to those in the sham group, and those in the NBF + ABT-263 (25 mg/kg) and NBF + ABT-263 (50 mg/kg) groups were significantly lower than those in the NBF group. The NBF + ABT-263 (50 mg/kg) group had lower SC, BUN, and diameters of the renal pelvis than the NBF + ABT-263 (25 mg/kg) group. The results were significantly different (*p* < 0.05).

### 3.2. ABT-263 treatment improved the kidney morphology of NBF rats

**Figure 2.**
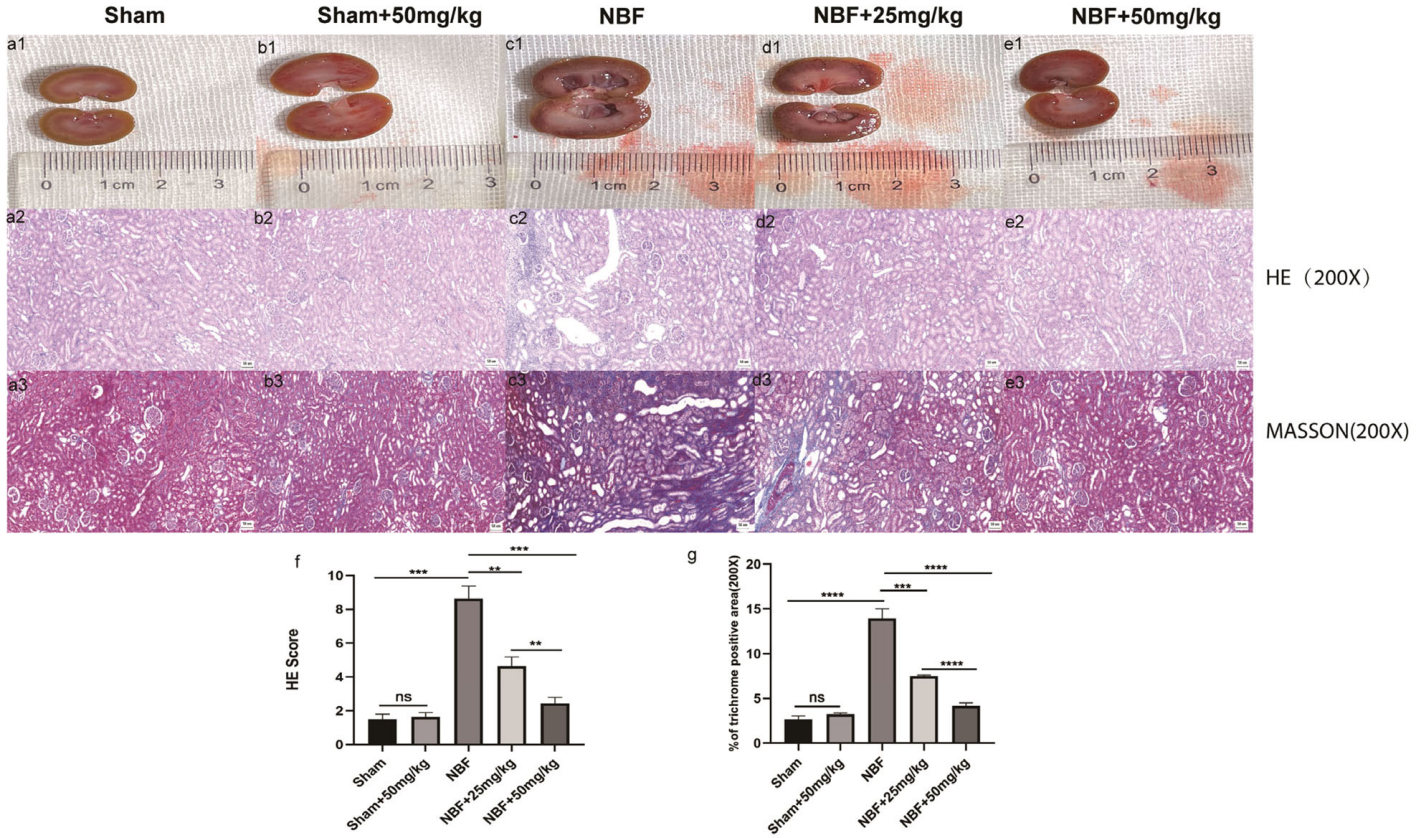
Staining of kidney tissue sections in each group.a1–e1 represent the general condition of the kidneys in the sham group, sham + ABT-263 group (50 mg/kg), NBF group, NBF + ABT-263 group (25 mg/kg), and NBF + ABT-263 group (50 mg/kg). a2–e2 represents the hematoxylin and eosin (HE) staining of the kidneys in each group. a3–e3 represent Masson staining of kidneys in each group. The kidneys in the sham and sham + ABT-263 group (50 mg/kg) groups were basically normal. The kidney damage and fibrosis in the NBF group were the most serious, followed by those in the NBF + ABT-263 group (25 mg/kg), and the kidney damage and fibrosis in the NBF + ABT-263 group (50 mg/kg) were greatly improved. f: Statistical histogram of renal HE staining scores in each group. g: Statistical histogram of renal Masson staining scores in each group. HE Scalebar = 50 μm. Masson Scale bar = 50 μm. Data are expressed as the mean ± SEM; ns *p* > 0.5, *p < 0.01, **p < 0.001, ***p < 0. 0001. All experiments were repeated three times.

HE and Masson staining showed that the kidneys of the sham and sham + ABT-263 (50 mg/kg) groups had normal morphology, no difference in score, and no difference in the area of renal fibrosis (Figure 2). The kidneys in the NBF group had abnormal morphology and infiltrated inflammation, the score was significantly higher, and the area of renal fibrosis was significantly increased compared to the sham and sham + ABT-263 (50 mg/kg) groups. The NBF + ABT-263 (25 mg/kg) and NBF + ABT-263 (50 mg/kg) groups had less abnormal kidney morphology, inflammatory infiltration, and inflammation as well as lower scores than the NBF and NBF + ABT-263 (25 mg/kg) groups. Additionally, the area of renal fibrosis was significantly reduced. Compared to the NBF + ABT-263 (25 mg/kg) group, the NBF + ABT-263 (50 mg/kg) group had less abnormal kidney morphology, inflammatory infiltration, and inflammation, and the score was lower than that of the NBF + ABT-263 (25 mg/kg) group. The area of renal fibrosis decreased, and the statistical results were significantly different (*p* < 0.05).

### 3.3. ABT-263 can improve the bladder function of NBF rats

**Figure 3.**
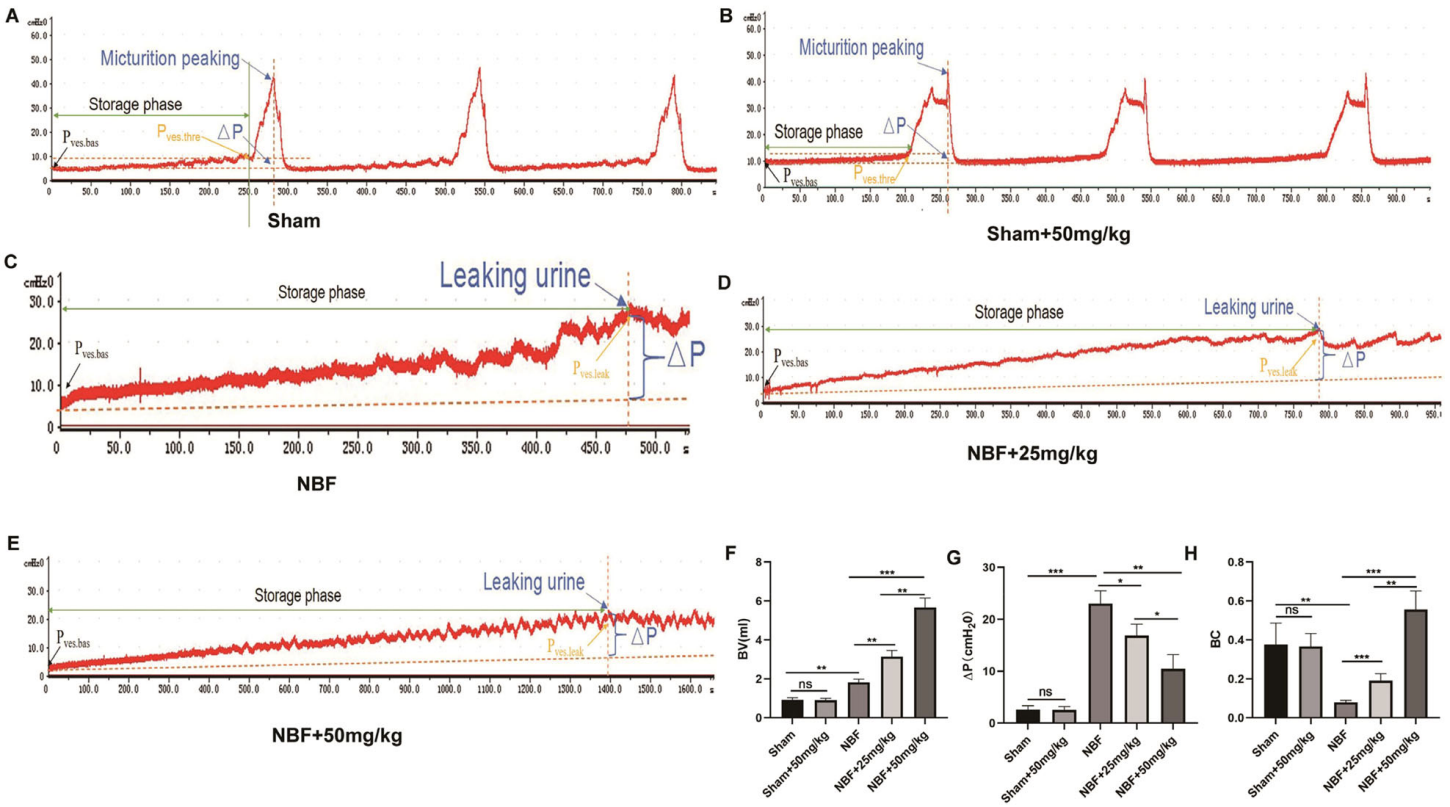
Cystometry in each group. (a) Representative bladder pressure curve of the sham group showing regular micturition. (b) Representative bladder pressure curve of the sham + ABT-263 (50 mg/kg) group showing regular micturition contractions. (c) The representative bladder pressure curve of the NBF group showing no micturition contraction when the intravesical pressure increased to reach the P_ves.leak_, and the rats experienced overflow incontinence. (d) Representative bladder pressure curve of the NBF + ABT-263 (25 mg/kg) group showing no micturition contraction when the intravesical pressure was increased to reach the P_ves.leak_. The rats experienced overflow incontinence, but their bladder volume was significantly larger than that in the NBF group. e: Representative bladder pressure curve of the NBF + ABT-263 (50 mg/kg) group showing no micturition contraction when the intravesical pressure was increased to reach the P_ves.leak_. The rats experienced overflow incontinence, but the bladder volume was significantly larger than that in the NBF + ABT-263 (25 mg/kg) group. f: The bladder volume (BV) of the NBF group was significantly higher than that of the sham group, while the BV of the NBF + ABT-263 (25 mg/kg) and NBF + ABT-263 (50 mg/kg) groups were significantly higher than that of the NBF group. Moreover, the BV of the NBF + ABT-263 (50 mg/kg) group was significantly higher than that of the NBF + ABT-263 (25 mg/kg) group. g: △p (= P_ves.leak_–P_ves.bas_) of the NBF group was significantly higher than that of the △p(=P_ves.thre_–P_ves.bas_) sham group, while the △p (= P_ves.leak_–P_ves.bas_) of the NBF + ABT-263 (25 mg/kg) and NBF + ABT-263 (50 mg/kg) groups were significantly lower than that of the NBF group. Moreover, △p of the NBF + ABT-263 (50 mg/kg) group was significantly lower than that of the NBF + ABT-263 (25 mg/kg) group. h: The bladder compliance (BC) of the NBF group was significantly lower than that of the sham group, while the BC of the NBF + ABT-263 (25 mg/kg) and NBF + ABT-263 (50 mg/kg) groups was significantly higher than that of the NBF group. Moreover, the BC content of the NBF + ABT-263 (50 mg/kg) group was significantly higher than that of the NBF + ABT-263 (25 mg/kg) group. Data are expressed as the mean ± SEM; *p < 0.01, **p < 0.001, ***p < 0. 0001.All experiments were repeated three times.

There was no significant difference in bladder capacity, detrusor pressure changes during filling, and BC between the sham and sham + ABT-263 (50 mg/kg) groups (Figure 3). Compared with the sham group, the NBF group had decreased bladder capacity and BC, and increased detrusor pressure during bladder filling. The NBF + ABT-263 (25 mg/kg) and NBF + ABT-263 (50 mg/kg) groups had larger bladder capacity and BC, and lower detrusor pressure during the bladder filling phase than that of the NBF group. Compared with the NBF + ABT-263 (25 mg/kg) group, the NBF + ABT-263 (50 mg/kg) group had a significantly increased bladder capacity and BC, and decreased detrusor pressure changes during the filling phase (*p* < 0.05).

### 3.4. ABT-263 can improve the bladder morphology of NBF rats

**Figure 4.**
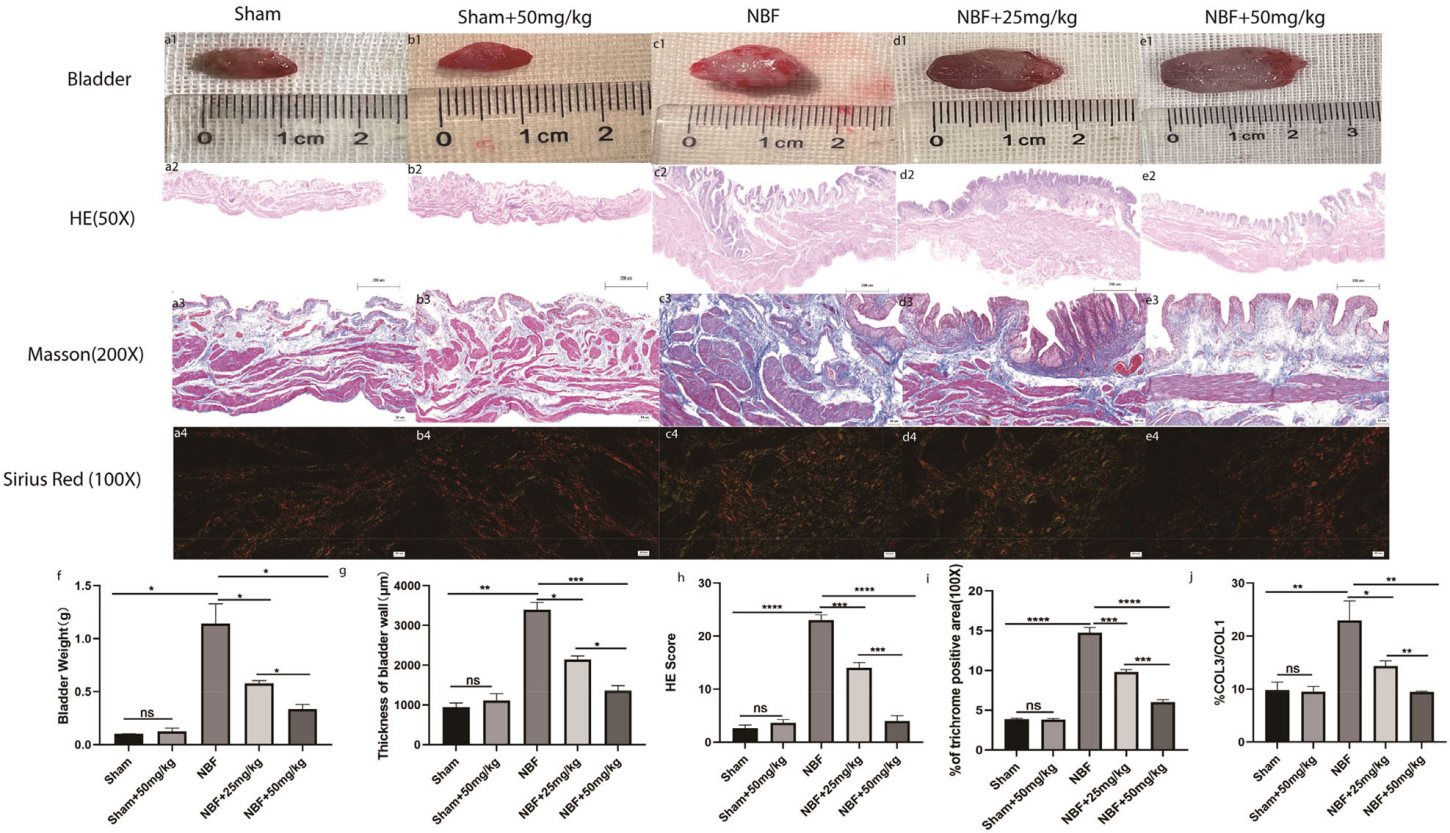
Staining of bladder tissue sections in each group. (a1–e1) represent the general condition of the bladder in the sham group, sham + ABT-263 group (50 mg/kg), NBF group, NBF + ABT-263 group (25 mg/kg), and NBF + ABT-263 group (50 mg/kg), respectively. (a2–e2) represents the matoxylin and eosin (HE) staining of the bladder in each group. (a3–e3) represent Masson staining of each bladder. a4–e4 represents Sirius red staining of the bladder in each group. (f) Statistical histogram of bladder weight in each group, which shows that bladder fibrosis was basically normal in the sham group and sham + ABT-263 group (50 mg/kg), the most serious in the NBF group, slightly improved in the NBF + ABT-263 group (25 mg/kg), and greatly improved in the NBF + ABT-263 group (50 mg/kg). (g) Statistics of the bladder HE staining score in each group histogram, which showed that bladder inflammation was basically normal in the sham and sham + ABT-263 (50 mg/kg) groups, the most serious in the NBF group, slightly improved in the NBF + ABT-263 group (25 mg/kg), and greatly improved in the NBF + ABT-263 group (50 mg/kg). (h) Statistical histogram of bladder fibrosis area in each group, which shows that bladder fibrosis was basically normal in the sham and sham + ABT-263 (50 mg/kg) groups, the most serious in the NBF group, slightly improved in the NBF + ABT-263 group (25 mg/kg), and greatly improved in the NBF + ABT-263 group (50 mg/kg). j: Statistical histogram of the Col3/Col1 ratios for each group. HE Scale bar = 200 μm. Masson Scale bar = 50 μm. Sirius red Scalebar = 100 μm. Data are expressed as the mean ± SEM; ns *p* > 0.5, *p < 0.01, **p < 0.001, ***p < 0. 0001. All experiments were repeated three times.

There were no significant differences in bladder weight, morphology score, fibrosis area, or col3/col3 ratio between the sham and sham + ABT-263 (50 mg/kg) groups (Figure 4). Compared with the sham group, the bladder weight, morphological score, fibrosis area, and col3/col11 ratio were all significantly increased in the NBF group (*p* < 0.05). The bladder weight, morphological score, fibrosis area, and col3/col3 ratio in the NBF + ABT-263 (25 mg/kg) and ABT-263 (50 mg/kg) groups were significantly decreased compared with those in the NBF group (*p* < 0.05). The bladder weight, morphological score, fibrosis area, and col3/co11 ratio of the ABT-263 (50 mg/kg) group were significantly lower than those of the NBF + ABT-263 (25 mg/kg group) (*p* < 0.05).

### 3.5. ABT-263 can reduce bladder fibrosis in NBF rats

**Figure 5.**
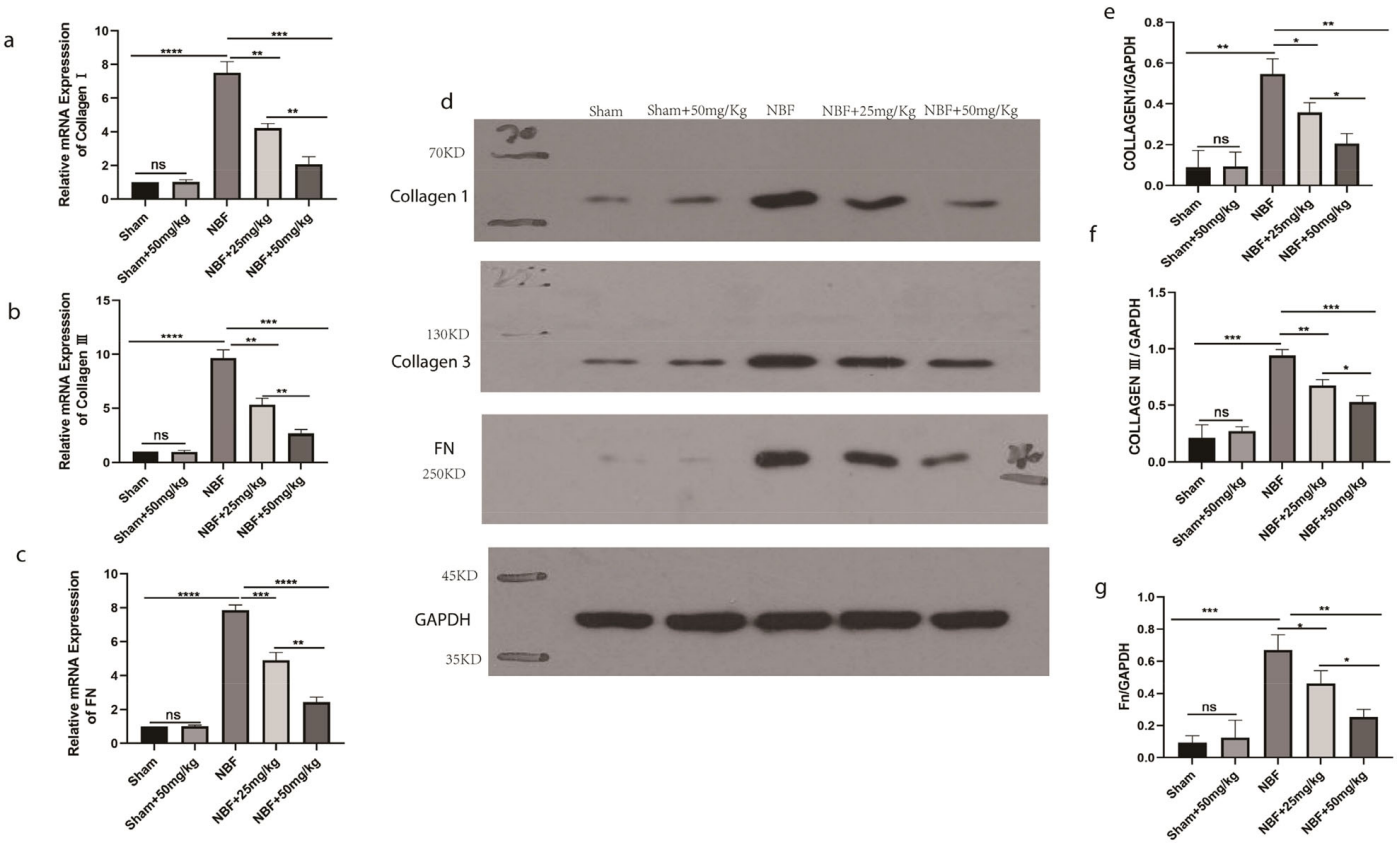
WB and qPCR results of col1, col3, and FN in the bladder tissues of different groups. (a–c) Statistical histogram of the relative ratio of col1, col3, and Fn mRNA in bladder tissue in the sham, sham + ABT-263 (50 mg/kg), NBF group, NBF + ABT-263 (25 mg/kg), NBF + ABT-263 (50 mg/kg) groups; d: WB in bladder tissue in sham, sham + ABT-263 (50 mg/kg), NBF, NBF + ABT-263 (25 mg/kg), and NBF + ABT-263 (50 mg/kg) groups, with GADPH as the internal reference protein. (e–g) Relative expression of col1, col3, and FN protein in bladder tissue in sham, sham + ABT-263 (50 mg/kg), NBF, NBF + ABT-263 (25 mg/kg), and NBF + ABT-263 (50 mg/kg) groups. The histogram of ratio statistics shows that bladder fibrosis were basically normal in the sham and sham + ABT-263 (50 mg/kg) groups, the most serious in the NBF group, slightly improved in the NBF + ABT-263 group (25 mg/kg), and greatly improved in the NBF + ABT-263 group (50 mg/kg). Data are expressed as the mean ± SEM; ns *p* > 0.5, *p < 0.01, **p < 0.001, ***p < 0.0001. All experiments were repeated three times.

There was no significant difference in the expression of col1, col3, and FN in the bladder tissue of the sham group compared to the sham + ABT-263 (50 mg/kg) group (Figure 5). The expression levels of col1, col3, and FN in the bladder tissue of the NBF group were significantly increased (*p* < 0.05) compared with those in the sham group, and those in the NBF + ABT-263 (25 mg/kg) and NBF + ABT-263 (50 mg/kg) group were significantly decreased compared with those in the NBF group. The level in the NBF + ABT-263 (50 mg/kg) group was significantly lower than that in the NBF + ABT-263 (25 mg/kg) group (*p* < 0.05).

### 3.6. ABT-263 can reduce bladder tissue expression of the anti-apoptotic protein BCL-xL in NBF rats

**Figure 6.**
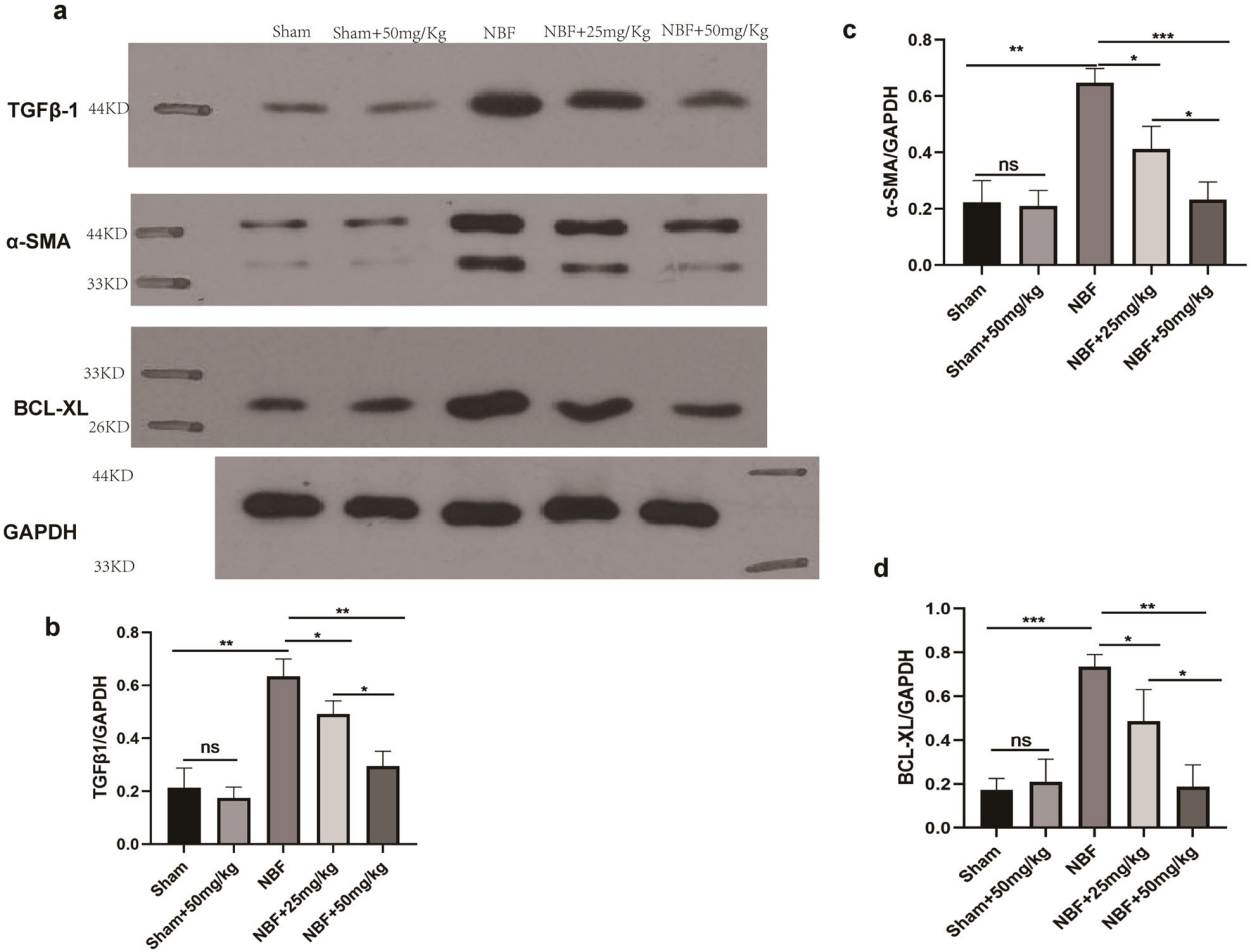
Protein expression of the TGF-β1/αSMA/BCL-XL signaling pathway in the bladder tissues of different groups. (a) Protein expression of TGF-β1, α-SMA, and BCL-xL in the sham, sham + ABT-263 (50 mg/kg), NBF, NBF + ABT-263 (25 mg/kg), and NBF + ABT-263 (50 mg/kg) groups. (b–d) The histogram of ratio statistics of protein expression of TGF-β1, α-SMA, and BCL-xL in bladder tissue shows that the TGF-β1/αSMA/BCL-xL signal pathway expression was normal in the sham and sham + ABT-263 (50 mg/kg) groups, had the highest overexpression in the NBF group, was improved in the NBF + ABT-263 group (25 mg/kg), and greatly improved in the NBF + ABT-263 group (50 mg/kg).ns *p* > 0.5, *p < 0.01,**p < 0.001, ***p < 0.0001.All experiments were repeated three times.

There was no difference in the protein expression of TGF-β1, α-SMA, and BCL-xL in the bladder tissue of the sham group compared to that of sham + ABT-263 (50 mg/kg) (Figure 6). The protein expression levels of TGF-β1, α-SMA, and BCL-xL in the bladder tissue of the NBF group were significantly increased compared with those in the sham group (*p* < 0.05). Those in the bladder tissue of the NBF + ABT-263 (25 mg/kg) and NBF + ABT-263 (50 mg/kg) groups were significantly decreased compared with those in the NBF group, and those in the bladder tissue of the NBF + ABT-263 (50 mg/kg) group were significantly decreased compared with those in the NBF + ABT-263 (25 mg/kg) group (*p* < 0.05).

### 3.7. ABT-263 can promote the apoptosis of rat primary fibroblasts

Compared with the TGF-β1 group, when the concentration of ABT-263 was increased to 10 µmol/L, the cell viability of primary bladder fibroblasts began to decrease, the apoptosis rate increased, the fluorescence intensity of red fluorescent α-SMA decreased, and the protein expression of cytochrome C (Cyto C) and cleaved caspase-3 began to increase.

**Figure 7.**
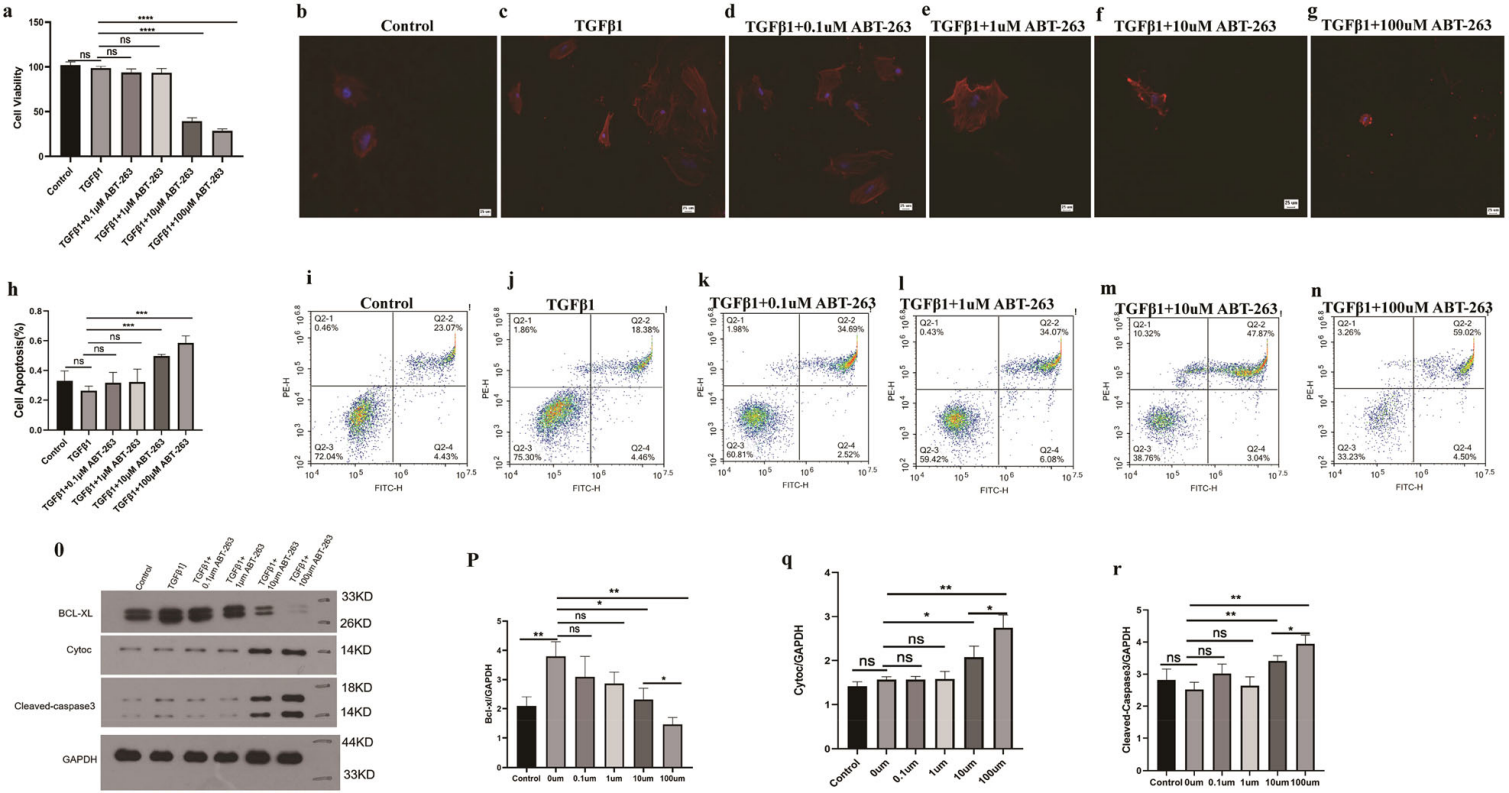
ABT-263 promotes apoptosis of primary bladder fibroblasts through the mitochondrial apoptosis pathway. Histogram of CCK8 statistics in primary bladder fibroblasts under different concentrations of ABT-263, which shows that when the concentration of ABT-263 increased to 10 µmol, cell viability began to deteriorate, and the cell viability rate deteriorated with increasing ABT-263 concentration–g: Changes in α-SMA fluorescence intensity of primary bladder fibroblasts under different concentrations of ABT-263, which shows that when the concentration of ABT-263 increased to 10 µmol, the α-SMA fluorescence intensity began to weaken, and the α-SMA fluorescence intensity decreased with increasing ABT-263 concentration. i–n: Flow cytometric representation of primary bladder fibroblasts treated with different concentrations of ABT-263.h: Histogram of apoptosis statistics of primary bladder fibroblasts under different concentrations of ABT-263 by representative flow cytometry, which shows that when the concentration of ABT-263 increased to 10 µmol, the cells began to undergo apoptosis, and the apoptosis rate increased with increasing ABT-263 concentration: Expression of mitochondrial pathway-related proteins in primary bladder fibroblasts treated with different concentrations of ABT-263.p–r: Histogram of BCL-xL, Cytoc, and cleaved caspase-3 proteins in primary bladder fibroblasts under different concentrations of ABT-263, which shows that when the concentration of ABT-263 increased to 10 µmol, the mitochondrial pathway began to activate, and the degree of activation increased with increasing ABT-263 concentration. Data are expressed as the mean ± SEM; ns *p* > 0.5, *p < 0.01, **p < 0.001, ***p < 0.0001. All experiments were repeated three times.

When the concentration of ABT-263 was increased to 100 µmol/L, the cell viability decreased, the apoptosis rate increased more notably (*p* < 0.05), and the statistical results differed. The protein expression of Cytoc and cleaved caspase-3 increased significantly (Figure 7).

**Figure 8.**
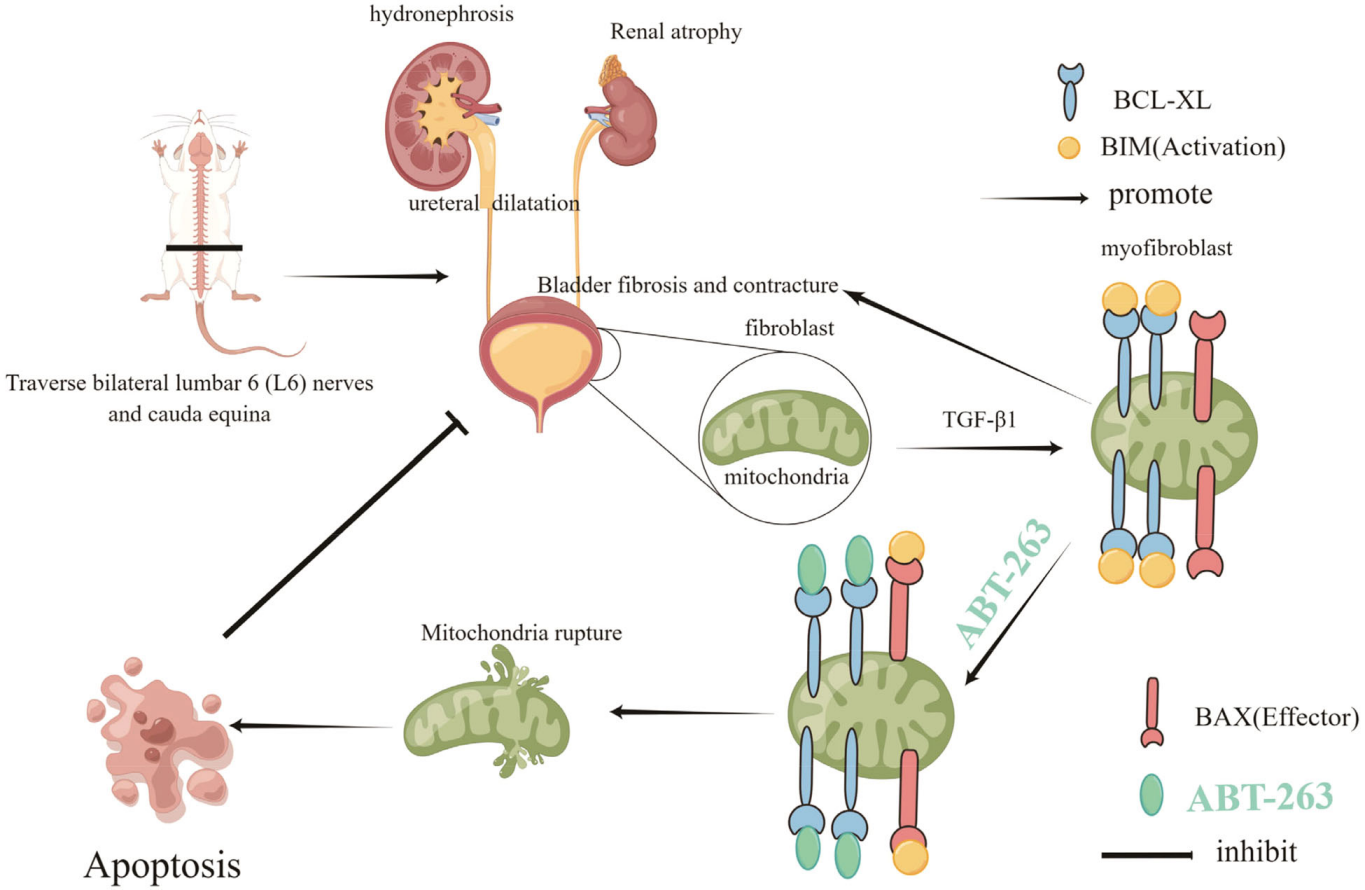
A sketch of the experiment.

The expression of BCL-xL in the TGF-β1 group was significantly higher than that in the blank control group. In fibroblasts, when the concentration of ABT-263 was increased to 10 µmol/L, BCL-xL expression began to decrease, and when the concentration of ABT-263 increased to 100 µmol/L, BCL-xL expression decreased further. A sketch of the experiment is shown in Figure 8.

## 4. Discussion

NB is an issue worldwide and there are currently no effective therapies. The main purpose of NB treatment should be to protect upper urinary tract function and maximize the life expectancy of patients [26].

The most common complication of NB is urinary tract infection [27]. The long-term effects of inflammatory factors promote the occurrence of epithelial tissue anaplastic and fibrosis, which in turn leads to the continuous differentiation of quiescent fibroblasts, epithelial cells, and circulating mononuclear macrophages into myofibroblasts [28]. It is well known that NBF decreases bladder compliance, increases bladder pressure, and induces UUPD. Therefore, controlling the progression of NBF is an important strategy to protect the upper urinary tract function of patients with NB [29].

Wang et al. [30] showed that a contractile bladder decreased BC, and increased detrusor leakage point pressure (DLPP) are major urodynamic risk factors for UUTD in children with NB. However, this study only showed the risk factors for UUTD and did not clarify the pathophysiology and molecular mechanisms involved in NBF. We established an animal model of NBF by amputating the bilateral lumbar 6 and cauda equina nerves in rats. Using this model, Chen et al. [31] found that the TGFβ/Smad signaling pathway in NBF rats is gradually activated over time, and bladder fibrosis is gradually aggravated; however, there is an effective lack of treatment measures. Although He et al. [24] found a protective effect with four weeks of ‘losartan’ treatment on bladder fibrosis and renal function, a long-term study of treatment observation after NBF is lacking.

The NB animal model at three months after the surgery is equivalent to the NB late stage, (i.e., bladder with severe fibrosis, low compliance, areflexia, and hydronephrosis). We used female rats because the Crede maneuver will more easily induce urination in female rats than in male rats. In addition, we used rats of the same sex to avoid the potential effect of sex on the experimental results.

It has been reported that ABT-263 is a BCL-2 family protein inhibitor produced during the clinical development of ABT-737 [32]. It binds to the BH3 domain of the BCL-2 protein located in the cytoplasm, resulting in BIM protein release [33] and triggers the release of cytochrome C(CytoC) in the mitochondria, causing the release of the apoptotic protein cleaved caspase-3, leading to cell apoptosis [33]. The present study demonstrated that ABT-263 increased CytoC and cleaved caspase-3 expression and decreased BCL-xL expression, indicating that the mitochondrial pathway might also involve in ABT-263-induced myofibroblasts apoptosis. Considering the anti-apoptotic capacity of myofibroblasts, which plays a crucial role in the progression of bladder fibrosis, is greatly increased, targeting myofibroblasts may be an effective strategy to alleviate NBF.

We started to perform ABT-263 drug intervention for four weeks on the rats after the successful establishment of NBF induced by sacral nerve injury. Compared with the NBF group, we found that the NBF rats treated with NBF + ABT-263 (25 mg/kg) significantly relieved UUTD, and SC and BUN also significantly decreased. HE staining showed that the morphology of the kidney tissue was significantly improved, and Masson staining showed that renal fibrosis was reduced. This improvement was more significant in the NBF + ABT-263 group (50 mg/kg). This indicates that ABT-263 alleviated UUTD in NBF rats. It is well known that increased intravesical pressure caused by bladder fibrosis is closely related to the progression of UUTD in NBF; therefore, we speculate that ABT-263 can alleviate bladder fibrosis in NBF rats.

Compared with the NBF group, cystometry of NBF rats showed increased BC and bladder capacity and decreased DLPP after NBF + ABT-263 (25 mg/kg) treatment, and these urodynamic parameters were improved more significantly in the NBF + ABT-263 (50 mg/kg) group, suggesting that ABT-263 may alleviate bladder fibrosis. In addition, compared with the NBF group, the bladder weight and the thickness of the bladder wall were reduced significantly after NBF + ABT-263 (25 mg/kg) treatment. HE staining showed that bladder epithelial metaplasia was alleviated and the degree of inflammatory infiltration was reduced, Masson staining also showed that bladder fibrosis was alleviated, and Sirius red staining showed that the ratio of collagen type col3/col3 decreased. The WB results also showed that Col1, Col3, and Fn in the bladder tissue of NBF rats were significantly reduced after NBF + ABT-263 (25 mg/kg) treatment, and the degree of fibrosis was significantly reduced in the NBF + ABT-263 (50 mg/kg) group than in the NBF + ABT-263 (25 mg/kg) group, indicating that fibrosis progression was effectively controlled after ABT-263 treatment. Compared with the NBF group, the WB results showed that the protein expression of TGF-β1, α-SMA, and BCL-xL in the bladder tissue of the NBF rats decreased after NBF + ABT-263 (25 mg/kg) and NBF + ABT-263 (50 mg/kg) treatment, which indicated that the anti-apoptotic ability of collagen fibre-producing cells in the bladder of NBF rat tissue was decreased after ABT-263 treatment.

We isolated primary bladder fibroblasts to explore the mechanistic role of ABT-263 in the treatment of bladder fibrosis. TGF-β1, which stimulates fibroblast differentiation into myofibroblasts, is often characterized by the expression of α-SMA protein and has a strong anti-apoptotic ability [34,35]. We first stimulated fibroblasts with TGF-β1 (10 ng/mL) for 24 h, and then added ABT-263 at concentrations of 0, 0.1, 1, 10, and 100 µmol/L. When the concentration of ABT-263 was increased to 10 µmol/L, the number of apoptotic cells increased, and the statistical results showed significant differences. In addition, immunofluorescence showed that the fluorescence intensity of α-SMA decreased at 10 µmol/L, and the annexin/PI experiment further confirmed that the number of apoptotic cells increased. The above experimental results showed that when the concentration of ABT-263 was increased to 10 µmol/L, apoptosis of bladder myofibroblasts was promoted. The WB results showed that when the concentration of ABT-263 was increased to 10 µmol/L, the protein expression of α-SMA and BCL-xL decreased, while that of Cytoc and cleavedcaspase-3 increased, indicating that ABT-263 promoted myofibroblast apoptosis through the mitochondrial apoptosis pathway. We speculate that this is because, under the stimulation of TGF-β1, primary bladder fibroblasts differentiate into myofibroblasts, which have a stronger anti-apoptotic ability. It is well known that there are only a few myofibroblasts in the bladder of one-week-old SD rats. The cells showing α-SMA positive expression indicate that the cells we used are most likely myofibroblasts. Considering that fibroblasts are static cells that cannot secrete fibro-protein and collagen, we used TGF-β1 to stimulate their transformation into activated myofibroblasts that can secrete fibrosis and collagen.

ABT-263 was identified in an earlier study by Tseet al. [32], who reported reversible thrombocytopenia with daily and escalating dose regimens in a dog animal model. Even an escalating dose regimen initially caused a platelet drop of approximately 50%; however, similar steady-state effects were observed with continued high-dose administration. Similarly, Shoemaker et al. [36] and Lock et al. [37] observed transient thrombocytopenia and lymphopenia *in vivo* in small cell carcinoma of lung and acute lymphoblastic leukemia xenograft models, respectively. They characterized this event as the rapid clearance of circulating platelets undergoing apoptosis in response to ABT-263 treatment. However, it is worth noting that the occurrence of thrombocytopenia is reversible.

It has been known that ABT-263 is a potent, orally bioavailable Bad-like BH3 mimetic (K(i)'s of <1 nmol/L for BCL-2, BCL-xL, and BCL-w), which disrupts BCL-2/BCL-xL interactions with pro-death proteins (e.g., Bim), leading to the initiation of apoptosis within 2 h post-treatment [32]. So far, it has mainly been used in cell (*in vitro*) and animal experiments (*in vivo*), although clinical trials have begun [37]. The doses of ABT-263 (25–100 mg/kg/d) are safe for a transplanted tumor mouse study, and 100 mg/kg ABT-263 caused rapid and complete tumor regression [32].

The dose selection of ABT-263 used in the present study mainly depends on research from Lagares et al. [17], who used an oral gavage at a maximum dosage of 100 mg/kg body weight in mice with skin fibrosis, and a publication from Pullarkat et al. [38], who reported that a recommended phase II dose of 50 mg ABT-263 (navitoclax) for adults and 25 mg for patients <45 kg were used with 400 mg adult-equivalent venetoclax to treat patients with relapsed or refractory acute lymphoblastic leukemia and lymphoblastic lymphoma[38].In the present study, two lower concentration gradients of 50 mg/kg and 25 mg/kg ABT-263 achieved satisfactory experimental expectations.

Based on the critical role of mitochondrial function in cellular health, a better understanding of the main molecular players in mitochondrial dysfunction might be decisive for the therapeutic control of fibrosis in NB [39]. Recently, Li et al. [16] found that chronic exposure to deltamethrin induces apoptosis of quail cerebrum neurons by promoting endoplasmic reticulum stress and mitochondrial dysfunction, indicating a relationship between apoptosis and mitochondria, and found that ABT-263 can promote the apoptosis of myofibroblasts through the mitochondrial apoptosis pathway, providing new insights into these areas. In addition, compared with the sham group, the sham + ABT-263 (50 mg/kg) group did not exhibit impaired renal or bladder function in rats. Therefore, the application of ABT-263 for the treatment of severe NBF is promising.

The spinal cord injuries that induce NB are severe. It would be ideal to simultaneously investigate the role of ABT-263 in a less severe NBF model. In addition, the pro-apoptotic role of ABT-263 in myofibroblasts in the NBF could also occur in the fibrotic kidney caused by the NBF bladder; therefore, the alleviation of renal fibrosis should not be only attributed to the pro-apoptotic role of ABT-263 in myofibroblasts in the bladder, and increasing bladder capacity and compliance which benefit the protection of renal function. Finally, the effect of ABT-263 seen on cell apoptosis in the present study is related to high concentrations which might not be achieved *in vivo*. These need to be investigated in the future.

In conclusion, this study confirmed that ABT-263 can promote apoptosis of bladder myofibroblasts through the mitochondrial pathway, alleviating the progression of NBF, increasing bladder capacity and compliance, and ultimately protecting the upper urinary tract.

## Data Availability

All data generated or analyzed during this study are available from the corresponding author upon reasonable request.

## References

[1] Liao L. Evaluation and management of neurogenic bladder: what is new in China? IJMS. 2015;16(8):18580–18600. 26266405 10.3390/ijms160818580 PMC4581261

[2] Stein R, Bogaert G, Dogan HS, et al. EAU/ESPU guidelines on the management of neurogenic bladder in children and adolescent part I diagnostics and conservative treatment. Neurourol Urodyn. 2020;39(1):45–57. 31724222 10.1002/nau.24211

[3] Panicker JN, Fowler CJ, Kessler TM. Lower urinary tract dysfunction in the neurological patient: clinical assessment and management. Lancet Neurol. 2015;14(7):720–732. 26067125 10.1016/S1474-4422(15)00070-8

[4] Gajewski JB, Schurch B, Hamid R, et al. An international continence society (ICS) report on the terminology for adult neurogenic lower urinary tract dysfunction (ANLUTD). Neurourol Urodyn. 2018;37(3):1152–1161. 29149505 10.1002/nau.23397

[5] Peyronnet B, Richard C, Bendavid C, et al. Urinary TIMP-2 and MMP-2 are significantly associated with poor bladder compliance in adult patients with spina bifida. Neurourol Urodyn. 2019;38(8):2151–2158. 31486131 10.1002/nau.24163

[6] Tiryaki S, Yagmur I, Parlar Y, et al. Botulinum injection is useless on fibrotic neuropathic bladders. J Pediatr Urol. 2015;11(1):27.e1–27.e4. 10.1016/j.jpurol.2014.08.009 25448589

[7] Li P, Liao L, Chen G, et al. Early low-frequency stimulation of the pudendal nerve can inhibit detrusor overactivity and delay progress of bladder fibrosis in dogs with spinal cord injuries. Spinal Cord. 2013;51(9):668–672. 23797568 10.1038/sc.2013.60

[8] Anumanthan G, Tanaka ST, Adams CM, et al. Bladder stromal loss of transforming growth factor receptor II decreases fibrosis after bladder obstruction. J Urol. 2009;182(4S):1775–1780. 19692014 10.1016/j.juro.2009.05.126 PMC4797651

[9] Kim KK, Sheppard D, Chapman HA. TGF-β1 signaling and tissue fibrosis. Cold Spring Harb Perspect Biol. 2018;10(4):a022293. 28432134 10.1101/cshperspect.a022293 PMC5880172

[10] Massagué J. TGFβ signalling in context. Nat Rev Mol Cell Biol. 2012;13(10):616–630. 22992590 10.1038/nrm3434 PMC4027049

[11] Schultz GS, Wysocki A. Interactions between extracellular matrix and growth factors in wound healing. Wound Repair Regen. 2009;17(2):153–162. 19320882 10.1111/j.1524-475X.2009.00466.x

[12] Fry CH, Kitney DG, Paniker J, et al. Fibrosis and the bladder, implications for function ICI-RS 2017. Neurourol Urodyn. 2018;37(S4):S7–S12. 10.1002/nau.23725 30133788

[13] Larson SA, Dolivo DM, Dominko T. Artesunate inhibits myofibroblast formation via induction of apoptosis and antagonism of pro-fibrotic gene expression in human dermal fibroblasts. Cell Biol Int. 2019;43(11):1317–1322. 31441159 10.1002/cbin.11220

[14] Wang W, Zhou PH, Xu CG, et al. Baicalein ameliorates renal interstitial fibrosis by inducing myofibroblast apoptosis invivo and invitro. BJU Int. 2016;118(1):145–152. 26178456 10.1111/bju.13219

[15] Lee JW, Oh JE, Rhee KJ, et al. Co-treatment with interferon-γ and 1-methyl tryptophan ameliorates cardiac fibrosis through cardiac myofibroblasts apoptosis. Mol Cell Biochem. 2019;458(1-2):197–205. 31006829 10.1007/s11010-019-03542-7 PMC6616223

[16] Li S, Wu P, Han B, et al. Deltamethrin induces apoptosis in cerebrum neurons of quail via promoting endoplasmic reticulum stress and mitochondrial dysfunction. Environ Toxicol. 2022;37(8):2033–2043. 35446475 10.1002/tox.23548

[17] Lagares D, Santos A, Grasberger PE, et al. Targeted apoptosis of myofibroblasts with the BH3 mimetic ABT-263 reverses established fibrosis. Sci Transl Med. 2017;9(420):eaal3765. 29237758 10.1126/scitranslmed.aal3765 PMC8520471

[18] Pan J, Li D, Xu Y, et al. Inhibition of bcl-2/xl with ABT-263 selectively kills senescent type II pneumocytes and reverses persistent pulmonary fibrosis induced by ionizing radiation in mice. Int J Radiat Oncol Biol Phys. 2017;99(2):353–361. 28479002 10.1016/j.ijrobp.2017.02.216 PMC6853175

[19] Ackler S, Mitten MJ, Chen J, et al. Navitoclax (ABT-263) and bendamustine ± rituximab induce enhanced killing of non-Hodgkin’s lymphoma tumours in vivo. Br J Pharmacol. 2012;167(4):881–891. 22624727 10.1111/j.1476-5381.2012.02048.x PMC3575786

[20] Walaszczyk A, Dookun E, Redgrave R, et al. Pharmacological clearance of senescent cells improves survival and recovery in aged mice following acute myocardial infarction. Aging Cell. 2019;18(3):e12945. 30920115 10.1111/acel.12945 PMC6516151

[21] Li YL, Wen JJ, Wen YB, et al. Reconstruction of bladder function and prevention of renal deterioration by means of end-to-side neurorrhaphy in rats with neurogenic bladder. Neurourol Urodyn. 2018;37(4):1272–1280. 29160569 10.1002/nau.23456

[22] Chen J, Jin S, Abraham V, et al. The bcl-2/Bcl-X(L)/bcl-w inhibitor, navitoclax, enhances the activity of chemotherapeutic agents in vitro and in vivo. Mol Cancer Ther. 2011;10(12):2340–2349. 21914853 10.1158/1535-7163.MCT-11-0415

[23] Chang HY, Havton LA. Differential effects of urethane and isoflurane on external urethral sphincter electromyography and cystometry in rats. Am J Physiol Renal Physiol. 2008;295(4):F1248–F1253. 18753298 10.1152/ajprenal.90259.2008 PMC2576142

[24] He YL, Wen JG, Pu QS, et al. Losartan prevents bladder fibrosis and protects renal function in rat with neurogenic paralysis bladder. Neurourol Urodyn. 2021;40(1):137–146. 33606304 10.1002/nau.24567

[25] Zhao MT, Chen H, Liu Q, et al. Molecular and functional resemblance of differentiated cells derived from isogenic human iPSCs and SCNT-derived ESCs. Proc Natl Acad Sci USA. 2017;114(52):E11111–E11120. 29203658 10.1073/pnas.1708991114 PMC5748177

[26] Fang H, Lu B, Wang X, et al. Application of data mining techniques to explore predictors of upper urinary tract damage in patients with neurogenic bladder. Braz J Med Biol Res. 2017;50(10):e6638. 28832768 10.1590/1414-431X20176638 PMC5561813

[27] Madero-Morales PA, Robles-Torres JI, Vizcarra-Mata G, et al. Randomized clinical trial using sterile single use and reused polyvinylchloride catheters for intermittent catheterization with a clean technique in spina bifida cases: short-term urinary tract infection outcomes. J Urol. 2019;202(1):153–158. 30916625 10.1097/JU.0000000000000244

[28] Wang J, Chen Y, Gu D, et al. Ketamine-induced bladder fibrosis involves epithelial-to-mesenchymal transition mediated by transforming growth factor-β1. Am J Physiol Renal Physiol. 2017;313(4):F961–F972. 28331066 10.1152/ajprenal.00686.2016

[29] Lu YT, Tingskov SJ, Djurhuus JC, et al. Can bladder fibrosis in congenital urinary tract obstruction be reversed? J Pediatr Urol. 2017;13(6):574–580. 29037864 10.1016/j.jpurol.2017.08.013

[30] Wang QW, Wen JG, Song DK, et al. Is it possible to use urodynamic variables to predict upper urinary tract dilatation in children with neurogenic bladder-sphincter dysfunction? BJU Int. 2006;98(6):1295–1300. 17034510 10.1111/j.1464-410X.2006.06402.x

[31] Chen Y, Ma Y, He Y, et al. The TGF-β1 pathway is early involved in neurogenic bladder fibrosis of juvenile rats. Pediatr Res. 2021;90(4):759–767. 33469184 10.1038/s41390-020-01329-x

[32] Tse C, Shoemaker AR, Adickes J, et al. ABT-263: a potent and orally bioavailable bcl-2 family inhibitor. Cancer Res. 2008;68(9):3421–3428. 18451170 10.1158/0008-5472.CAN-07-5836

[33] Mérino D, Khaw SL, Glaser SP, et al. Bcl-2, bcl-x(L), and bcl-w are not equivalent targets of ABT-737 and navitoclax (ABT-263) in lymphoid and leukemic cells. Blood. 2012;119(24):5807–5816. 22538851 10.1182/blood-2011-12-400929 PMC3382939

[34] Islam SS, Mokhtari RB, El Hout Y, et al. TGF-β1 induces EMT reprogramming of porcine bladder urothelial cells into collagen producing fibroblasts-like cells in a Smad2/Smad3-dependent manner. J. Cell Commun. Signal. 2014;8(1):39–58. 24338442 10.1007/s12079-013-0216-4 PMC3972392

[35] Hinz B, Lagares D. Evasion of apoptosis by myofibroblasts: a hallmark of fibrotic diseases. Nat Rev Rheumatol. 2020;16(1):11–31. 31792399 10.1038/s41584-019-0324-5 PMC7913072

[36] Shoemaker AR, Mitten MJ, Adickes J, et al. Activity of the bcl-2 family inhibitor ABT-263 in a panel of small cell lung cancer xenograft models. Clin Cancer Res. 2008;14(11):3268–3277. 18519752 10.1158/1078-0432.CCR-07-4622

[37] Lock R, Carol H, Houghton PJ, et al. Initial testing (stage 1) of the BH3 mimetic ABT-263 by the pediatric preclinical testing program. Pediatr. Blood Cancer. 2008;50(6):1181–1189. 18085673 10.1002/pbc.21433

[38] Pullarkat VA, Lacayo NJ, Jabbour E, et al. Venetoclax and navitoclax in combination with chemotherapy in patientswith relapsed or refractory acute lymphoblastic leukemia and LymphoblasticLymphoma. Cancer Discov. 2021;11(6):1440–1453. 33593877 10.1158/2159-8290.CD-20-1465 PMC9533326

[39] Abate M, Festa A, Falco M, et al. Mitochondria as playmakers of apoptosis, autophagy and senescence. Semin Cell Dev Biol. 2020;98:139–153. 31154010 10.1016/j.semcdb.2019.05.022

